# Targeting Unmet Clinical Needs in the Treatment of Alcohol Use Disorder

**DOI:** 10.3389/fpsyt.2022.767506

**Published:** 2022-06-09

**Authors:** Falk W. Lohoff

**Affiliations:** Section on Clinical Genomics and Experimental Therapeutics, National Institute on Alcohol Abuse and Alcoholism, NIH, Bethesda, MD, United States

**Keywords:** alcohol, addiction, treatment, Personalized and Precision Medicine (PPM), mood disorder

## Abstract

Alcohol Use Disorder (AUD) is a chronic psychiatric disorder marked by impaired control over drinking behavior that poses a significant challenge to the individual, their community, the healthcare system and economy. While the negative consequences of chronic excessive alcohol consumption are well-documented, effective treatment for AUD and alcohol-associated diseases remains challenging. Cognitive and behavioral treatment, with or without pharmaceutical interventions, remain the most commonly used methods; however, their efficacy is limited. The development of new treatment protocols for AUD is challenged by difficulty in accurately measuring patterns of alcohol consumption in AUD patients, a lack of a clear understanding of the neuropsychological basis of the disorder, the high likelihood of AUD patients relapsing after receiving treatment, and the numerous end-organ comorbidities associated with excessive alcohol use. Identification and prediction of patients who may respond well to a certain treatment mechanism as well as clinical measurement of a patient's alcohol exposure are bottlenecks in AUD research which should be further addressed. In addition, greater focus must be placed on the development of novel strategies of drug design aimed at targeting the integrated neural pathways implicated in AUD pathogenesis, so that next-generation AUD treatment protocols can address the broad and systemic effects of AUD and its comorbid conditions.

## Introduction

Alcohol use disorder (AUD) is a common chronic disorder that is estimated to account for approximately 5% of the global disease burden (1, 2), an estimated 3.8% of global deaths (3), and which is associated with 88,000 deaths annually in the United States (4). AUD is also associated with several psychiatric and physical comorbidities and represents a high cost to society, estimated at ~$250 billion a year in the United States (5). The 12 month prevalence for AUD ranges from 5 to 14%, (6–8) and despite this high occurrence, significant unmet clinical needs exist in the management of AUD. Current medical treatment protocols have only limited efficacy in reducing the burden of the disorder. Further, AUD is a highly comorbid disorder, with strong associations between other psychiatric and downstream physical conditions. Treatment of AUD must therefore incorporate treatment for the systemic effects of the disorder and not simply the behavioral aspects of addiction or dependence. Addressing these critical needs is important to reduce overall illness associated morbidity and mortality. This article will succinctly review and summarize key unmet needs in AUD.

## Current Treatment Options for Alcohol Use Disorder

AUD is commonly treated with cognitive or behavioral interventions, pharmacological treatment, or a combination of these. The U.S. Food and Drug Administration (FDA) has approved three medications for the treatment of AUD expressly: disulfiram, acamprosate, and naltrexone, including an injectable form of naltrexone. Additionally, several drugs have been used off-label to treat AUD, such as topiramate, gabapentin, baclofen, ondansetron, and varenicline, among others (9). While these drugs have been used to treat AUD, and some patients do respond well to treatment, their efficacy is limited and none have been demonstrated to systematically and dramatically outperform placebo in reducing AUD symptoms, with many studies reporting a positive effect finding statistically significant but small effect sizes (10–13). Some studies have suggested that topiramate might outperform placebo in reducing drinking and craving, but the effect was minimal and not indicative of significant clinical potential (12, 14). The best support for effective pharmacological treatment of AUD exists currently for naltrexone and acamprosate, although meta-analyses of randomized double-blind placebo-controlled trials (RCTs) of those drugs found that in 40% to 70% of individuals taking either medication, there were no measurable positive outcomes, a finding not unusual for psychiatric mediations (12, 13, 15–17).

AUD patients' response to pharmacological treatment is also considerably heterogeneous, and clinical, environmental, genetic, and social factors can all contribute to variance in drug response in patients. Patients can exhibit varying degrees of clinical response and side effects, even to the same dose of the same drug, and some patients may respond very well to one treatment protocol but poorly to another. How success is defined in the context of pharmacological treatment also impacts the reported efficacy of different treatments. For instance, certain drugs may promote abstinence from drinking, while others may reduce the incidence of heavy drinking or end-organ comorbidities (18). Identifying what external factors may mediate patients' response to specific medicines remains a key challenge in the development of efficacious treatment for AUD.

In addition to pharmacological treatment, several options exist for psychotherapy or non-pharmacological treatments, such as individual psychotherapy, Cognitive-Behavioral Therapy (CBT), and 12-step facilitation groups including Alcoholics Anonymous (AA). Pharmacological and non-pharmacological interventions which have been assessed for use in treating AUD are summarized in Table 1. These interventions can be administered at the individual or group level and can help address problematic drinking by targeting maladaptive thought and behavior patterns or environmental triggers. Overall, studies have found psychological interventions for AUD to be somewhat effective compared to nonspecific controls, but also generally report that effect sizes are small, they do not work for all patients, and are no more effective than current pharmacological options (19, 20).

**Table 1:** Summary of currently available pharmacological and psychosocial interventions of AUD.

**Pharmacological Interventions**	**FDA approved for AUD**	**Clinical summary**	**References**
Disulfiram	Yes	Disulfiram exhibits an antidipsotropic effect characterized by nausea, tachycardia, and flushing. This drug works to decrease drinking through these severe physical symptoms.	(9, 21, 22)
Naltrexone	Yes	Naltrexone is a mu opioid antagonist that reduces reward reinforcement and alcohol craving.	(12, 13, 15, 22–25)
Injectable naltrexone	Yes	Injectable Naltrexone is an intramuscular gluteal injection that has been shown to increase patient compliance and minimize nonadherence due to its monthly administration as opposed to a daily ingestion. By bypassing first-pass metabolism, the injection also yields a more consistent blood level of Naltrexone.	(9)
Acamprosate	Yes	Acamprosate is a glutamate antagonist that promotes abstinence by normalizing the hyperglutamatergic state developed from continued alcohol dependence. It is thought to balance the glutamatergic and GABAergic systems associated with chronic alcohol exposure and alcohol withdrawal and target relief craving.	(12, 17, 22)
Topiramate	No	Topiramate is FDA-approved to treat epilepsy and migraines but can also be prescribed to treat AUD. Topiramate works by diminishing craving.	(12, 14, 22)
Gabapentin	No	Gabapentin is originally meant to treat epilepsy and neuropathic pain. It has been prescribed to treat AUD because it is a GABA agonist and glutamate antagonist.	(9)
Baclofen	No	Baclofen is a GABA_B_ receptor agonist that is FDA-approved to treat spasticity from multiple sclerosis. Baclofen has been associated with sustained abstinence; it is also linked to adverse effects like sedation, numbness, and slurred speech.	(9, 12, 18)
Ondansetron	No	Ondansetron is FDA-approved for treating nausea in chemotherapy. It is a 5-HT_3_ receptor antagonist that reduces the release of dopamine and has been prescribed to reduce alcohol consumption in patients with AUD.	(9)
Varenicline	No	Varenicline is a partial α4β2 nicotinic acetylcholine receptor (nAChR) agonist originally prescribed for nicotine dependence. Because of the comorbid reward pathways between nicotine use disorder and alcohol use disorder, Varenicline sems promising as a potential treatment route for AUD.	(9)
Nalmefene	No	Nalmefene is an antagonist at the mu and delta opioid receptors and a partial agonist at the kappa opioid receptor. It is approved for decreasing alcohol consumption by reducing craving in Europe but not in the United States. One meta-analysis illustrated that Nalmefene was associated with a reduction in binge drinking and total alcohol consumption.	(12)
Selective serotonin reuptake inhibitors and serotonin-related drugs	No	Selective serotonin reuptake inhibitors (SSRIs) are FDA-approved for the treatment of depression and anxiety disorders – like Sertraline. Given that AUD is often comorbid with depression and anxiety, SSRIs present a promising avenue as a treatment option.	(26–29)
**Provider-based and psychosocial interventions**		**Clinical Summary**	
Alcoholics anonymous		Alcoholics Anonymous (AA) is a self-supporting, informal society whose purpose lies in staying sober and helping others achieve sobriety. This fellowship is a purely nonprofessional social intervention where members find strength in each other to reach and maintain sobriety.	(30)
12-step facilitation		12-Step Facilitation (TSF) covers 12 weekly sessions that encourages involvement in AA through understanding, acceptance, and engagement. It is an individual therapy and is meant to promote long-term abstinence.	(30)
Motivational enhancement therapy		Motivational Enhancement Therapy (MET) works by accentuating the motivation and commitment to change. In MET, therapists work with the patient to help him/her recognize the problems consequent to his/her drinking habits in an empathetic, cooperative, and nonconfrontational manner.	(31)
Cognitive behavioral therapy		Cognitive Behavioral Therapy (CBT) enables the patient to come to terms with his/her feelings and behaviors associated with excessive alcohol consumption. The therapist teaches the patient coping skills to handle cravings and triggers and works with the patient to develop relapse prevention plans.	(19, 20)
Brief interventions		Brief Interventions (BIs) are single, sixty-minute sessions with a health-care professional. Meta-analyses have shown that in individuals with mild AUD, BIs are powerful in reducing alcohol consumption. However, in individuals with moderate-to-severe AUD, BIs are found ineffective, as these patients require more long-term solutions.	(32)
Cue-exposure therapy		Cue-Exposure Therapy (CET) is a type of classical conditioning therapy whereby the patient is repeatedly exposed to alcohol-related stimuli without actually consuming alcohol. The goal of CET is to decrease craving and increase self-efficacy for coping with strong desires to drink in high-risk contexts.	(33, 34)
Aversion therapy		In Aversion Therapy, alcohol is repeatedly paired with an unpleasant stimulus like an electric shock or emetic drug to condition the patient to associate alcohol consumption with negative feelings/thoughts.	(35, 36)
Mindfulness-based therapies		Mindfulness-based therapies center on present-day awareness and nonjudgmental perceptions on one's current state. In AUD, it is used to handle cravings and prevent relapse. One meta-analysis demonstrated that mindfulness-based therapies had significant effects on the reduction of craving.	(37)

Behavioral interventions can be used as standalone treatment or in conjunction with pharmacological treatment, and while there is some evidence for their efficacy as a standalone treatment, research suggests that the greatest efficacy results from a combination of pharmacological and non-pharmacological treatment (19, 38, 39). The COMBINE study recruited 1383 recently alcohol-abstinent patients with primary alcohol dependence diagnoses and found that patients receving naltrexone, behavioral interventions, or both fared better on drinking outcomes, while acamprosate was ineffective with or without cognitive intervention (23). Interestingly, placebo pills and meetings with healthcare professionals both had a greater effect on drinking behavior than cognitive-behavioral approaches alone. The COMBINE study highlights the immense variation in responses to both behavioral and pharmacological interventions for AUD, and underscores the importance of finding new, highly effective medications that work for large groups of patients. While some medication may be effective for certain patients, it is clear that current pharmacological interventions cannot address the immense heterogeneity in the clinical presentation of AUD patients.

## The Need to Get Specialized Treatment and Access to Care

Despite the high prevalence and associated adverse effects of AUD, less than 20% of individuals with the disorder get any treatment, and of those <10 percent (i.e., 2% overall) get pharmacological treatment. Only a fraction of patients receive subspecialty treatment (9, 40). Treatment for AUD frequently consists of specialized cognitive, behavioral, or pharmacological interventions, oftentimes in combinations (Table 1). However, current forms of AUD treatment have often shown limited success in randomized controlled trials (10, 11). It should be noted, however, that blinding factors may play a role in the outcomes of these studies; disulfiram, for example, was associated positively with abstinence and negatively with relapse in open-label studies (9). A meta-analysis of randomized controlled trials of disulfiram found that open-label studies alone reported significant effects compared to placebo, possibly due to patients' fear of adverse reactions (21).

In addition, most individuals with AUD first present to their general practitioner, but often do not have access to specialized addiction treatment (41, 42). This represents a missed opportunity to provide the best specialized care if clinically indicated. The National Institute of Alcohol Abuse and Alcoholism (NIAAA) is addressing this important aspect of access to care by providing information on available treatment options. The NIAAA Alcohol Treatment Navigator is an online tool that guides individuals toward evidence-based treatment from providers near their area; this is especially relevant because access to care is one of the main barriers to AUD treatment access (https://alcoholtreatment.niaaa.nih.gov/). The American College of Academic Addiction Medicine also offers fellowship programs and other training resources for clinicians to become trained and certified in addiction medicine. As AUD grows in prevalence and severity, it is all the more crucial for physicians to be able to recognize and effectively treat this patient population.

## The Need to Identify Which Patient Will Respond to Which Treatment

Like many other substance abuse and psychiatric disorders, treatment response to medication for AUD is heterogeneous; individual patients can exhibit divergent treatment responses and side effects when treated with the same drug, and even at the same dosage, with some patients responding favorably to one treatment but not another. The inability to identify prior to initiating treatment who will respond to and tolerate a chosen AUD medication often leads to a prescribing process of trial-and-error, adverse therapeutic outcomes, and unnecessary prolongation of AUD disease activity, which can promote the progression of comorbidities and negative health outcomes.

The promise of personalized medicine and pharmacogenomics brings the possibility of identifying *a priori* which drugs might work best for a specific individual with AUD (43). Pharmacogenomics refers to the testing of genetic markers (single nucleotide polymorphisms – SNPs) that can predict treatment response and adverse event profiles by altering pharmacokinetic and pharmacodynamic processes. While several products in the psychiatric space have claimed to offer improved targeting of initial medication choices based on pharmacogenetic testing profiles, current data in AUD does not support routine pharmacogenetic testing (44, 45). More AUD-specific genomic investigation is needed to determine whether any SNP drug targets have potential for targeted medications, which could allow for new, gene-specific mechanisms of pharmacological interventions.

## The Need for Greater Efficacy Across Clinician and Patient Reported Outcomes

Treatments for AUD rely on accurate measures of alcohol consumption and drinking behaviors. Researchers have broadly utilized retrospective self-report evaluations like the Timeline Follow-Back (TLFB) to measure alcohol intake and patterns. While the TLFB has been linked to valid assessments of short and long-term estimations of alcohol consumption, self-report evaluations tend to systematically underestimate drinking due to the unreliability of human memory, namely recall bias (46). Considering that participants using TLFB evaluations consistently underreport their drinking, the field should move toward more reliable objective measures to assess alcohol consumption (46). For example, daily drinking diaries can be used to decrease the amount of time for which participants must recall their drinking behavior relative to longer-term measurements such as the TLFB, which may improve participants' ability to accurately recall their drinking behavior. The advance of modern consumer technology and the proliferation of mobile devices such as smartphones has also enabled real-time self-report methods that can account for situational variables and may be more accurate than traditional memory-based recall measures such as the TLFB. These methods are part of the collective framework known as Ecological Momentary Assessment (EMA), which incorporates technology into self-report measures to allow for more accurate reports of drinking patterns in the short term (47). However, while these methods may be more valid than traditional recall questionnaires, they are still subject to inherent limitations of self-report assessments, including behavioral reactivity (i.e., the “observer effect”) and a reliance on participant compliance (47). Real-time daily assessments for alcohol consumption using biosensors that can continuously monitor alcohol levels may alleviate these concerns, and should be further explored as an objective assessment of drinking behavior (48). The use of objective alcohol consumption level monitoring could also improve efficiency and predictive validity in standard medication testing paradigms by providing detailed consumption data for outcome measures (48).

## The Need for New Medication Development Capable of Attenuating Domains of AUD

The heterogenic nature of AUD requires a multi-disciplinary treatment approach to address the many unmet medical needs among those with AUD. Current pharmacotherapies are often characterized by limited efficacy and fail to show promising results in clinical use (49). Further, the considerable heterogeneity of AUD patients makes it especially challenging to design a single drug capable of treating AUD patients as a whole. This inability of existing medications to effectively treat AUD illustrates the need to pursue new neuroscience-based pharmacotherapies that target the various neural and molecular pathways of addiction individually (49). These strategies may yield drugs which can target specific neurological AUD characteristics to more effectively treat individual patients.

Better understanding of the neurofunctional domains underlying AUD by assessing severity, modeling heterogeneity, predicting course, and targeting treatments can lead to improved medications (50). Previous studies have mainly focused on single domains of function such as cognitive control or social/emotional processing. A comprehensive framework can capture essential factors of neuropsychological functioning in people with varying degrees of AUD, which is crucial for developing effective treatments.

Kwako et al. (50) developed the Addictions Neuroclinical Assessment (ANA), a neuroscience-based framework that proposes that 3 domains are implicated in substance use disorders: incentive salience, negative emotionality, and executive function. These 3 neurofunctional factors are interrelated because of their shared underlying neural circuitry and shared genetic and environmental risk factors.

The *incentive salience* construct refers to the psychological “wanting” that is driven by both physiological factors and learned associations about a reward cue, prompting compulsive habits (51). Cue exposure is related to increased craving and changes in the neural reward system, facilitating chemical dependence (52). In AUD, factors like depression, trait anxiety scores, and items from the Obsessive Compulsive Drinking Scale load onto the incentive salience domain (50).

The *negative emotionality* domain, which proposes that a decrease in negative affect drives excessive alcohol consumption, encompasses markers including increased scores in neuroticism, aggression, trait anxiety, and overall difficulties in emotion regulation, which are often exhibited by AUD patients (50). As in the incentive salience factor, craving also maps onto negative emotionality; the desire to avoid withdrawal is mediated by the associated negative affect. More specifically, it is believed that individuals with AUD drink for the sole purpose of avoiding the negative emotionality states associated with withdrawal shifting from pleasant feelings following consumption to feelings of relief instead (52).

Finally, the *executive function domain* encompasses the higher-order mental processes involved in cognitive control and future planning. Subdomains in this field relevant for addiction include items like response inhibition, working memory, impulsivity, and premeditation (50, 51). Inefficient executive cognitive functioning puts the maintenance of abstinence at risk, thereby increasing the likelihood of relapse.

Using an extensive range of scales and neuropsychological tests, it was found that these 3 neurofunctional domains differ between individuals with and without AUD, which further accentuates the pertinence of these factors for addiction (50). Other authors have analyzed the ANA framework and found that it can inform alcohol-specific outcomes and treatments; classifying patient groups using ANA-like qualitative classifications can serve as a useful mechanism for predicting drug efficacy (53). The negative functionality domain, in particular, was associated with drinking intensity at 12 months and coping motives at 6 months post-treatment for AUD (54). While more work is needed to conclude the extent to which the ANA framework can effectively predict treatment outcomes or identify treatment responders or nonresponses, it is a useful tool for informing hypotheses for ongoing drug and clinical research.

At present, medication development for AUD is marked by slow pace and high costs due to the failure of many compounds to succeed in clinical trials. Despite exploring potential molecular targets, many studies report little to no effect sizes using these medications. Therefore, novel strategies are needed to identify new treatments that address how the target interacts with other pathways and mechanisms. One such method focuses on the integration of biomolecular and cellular networks that are imperative for detecting multiple targets that drive AUD (55). By evaluating how these networks are connected in the neurocircuits integral to the underlying domains of AUD, researchers can formulate better therapies through a multi-disciplinary approach.

## The Need for Treatment With Sustained Therapeutic Effects

Chronic AUD is a progressive neurodegenerative disease with no definitive cure. Even with the few approved psychotropic medications, many people still suffer from AUD due to factors like social stigma, expense, and transient effects of currently available pharmacotherapy. For instance, a multivariate meta-analysis of 41 pharmacotherapy trials conducted from 1992 to 2009 found that the effect size for naltrexone, one of the FDA approved drugs, has steadily diminished in promoting abstinence and reducing heavy drinking since the earliest studies (56).

Some studies have found that pharmacological interventions are effective in reducing drinking behavior in AUD patients, with certain medications generally promoting certain improved outcomes. The Alcohol Clinical Trials Initiative (ACTIVE) workgroup and its members have reviewed clinical trials of AUD medications and found that some drugs are effective for certain patient populations (57). For instance, disulfiram has been found by some open-label studies to reduce drinking, and while the results of clinical trials investigating acamprosate have been mixed, it may be more effective in preventing relapse among detoxified patients than reducing drinking for general AUD patients (22). Similarly, naltrexone may be more effective in reducing relapse to heavy drinking than in promoting abstinence (22, 24, 25, 58). Further, while these medications have been found to help reduce AUD symptoms in some people and trials, no medication has been shown to work consistently and with large effect sizes across patient groups; future drug discovery efforts should seek to identify novel compounds and drug targets with large effect sizes and which are easier to use clinically (55, 58).

Relapse also complicates AUD treatment; studies of relapse rates in AUD patients and in studies of different interventions have yielded varying estimates on the prevalence and significance of relapse. Some studies indicate that between 20 and 80% of individuals who receive treatment and experience short-term remission are expected to relapse long-term (59). Other authors have argued that relapse is not absolute; the rate of reported relapse is substantially heterogeneous and can be influenced by demographics, framing, and interpretation of data (60, 61). Moreover, many study designs, particularly randomized controlled trials, do not typically assess long-term outcomes, such as total reduction in drinking behavior over several years and improvement of comorbid symptoms. Further research is needed to clearly establish the role of current pharmacological interventions in treating AUD in the long term. With such high relapse-rates, AUD is in critical need of effective and long-lasting therapeutic interventions so that people who do pursue treatment can continue to manage their condition after the initial treatments have run their course.

## The Need to Address Psychiatric Co-Morbidities

Besides being a complex substance use disorder itself, AUD is highly comorbid with other psychiatric disorders, including major depressive disorder (MDD) and anxiety disorders (62–64). A diagnosis of MDD or an anxiety disorder increases the likelihood of developing AUD (65) and common underlying biological and/or genetic factors are suspected to play a role in these relationships (66, 67). Identification of common predispositional genetic factors is crucial for the development of novel treatments for AUD, MDD, and/or anxiety (26).

One-third of AUD individuals also exhibit depressive symptoms (27). Selective serotonin reuptake inhibitors (SSRIs) are widely used to treat depression, and while clinical trials have examined their efficacy in treating comorbid AUD, these trials have failed to find a clinically significant effect. Sertraline, an FDA-approved SSRI for the treatment of depression, was associated with lowered alcohol consumption compared to placebo, but there was no difference in other drinking measures (28). A later study found no significant effect of sertraline on depressive symptoms or alcohol consumption (68). However, it has been reported that a combination of sertraline and naltrexone may have a significant effect on AUD-depression comorbidity; a treatment group receiving both medications exhibited a greater rate of abstinence from alcohol, greater time to relapse in heavy drinking, less depressive symptoms, and fewer adverse events compared to other treatment groups (69). The presentation of AUD patients varies considerably due to individual-level differences, and combining medications that target different domains of neuropsychiatric impairment may increase the therapeutic effect of a medication regiment while also allowing for effective treatment of a wider range of patients.

Comorbid anxiety disorders, including generalized anxiety disorder (GAD), panic disorder (PD), social phobia, specific phobia, post-traumatic stress disorder (PTSD), and obsessive-compulsive disorder (OCD), occur in approximately 5 to 30% of AUD patients. Conversely, among anxiety disorder patients, the prevalence of AUD ranges from 7 to 10%, with approximately 50% of patients using alcohol to self-medicate their anxiety symptoms (29). Further, a diagnosis of an anxiety disorder predicts development of AUD in patients non-pathologically misusing alcohol, and AUD diagnosis predicts GAD and SP diagnoses and onset of PD (29). Studies of sertraline in comorbid PTSD/AUD patients found that sertraline may have a limited effect on a small subset of patients, but could not conclude an overall beneficial effect (70). Further studies failed to find significant reduction in comorbid symptoms in patients treated with SSRIs or norepinephrine reuptake inhibitors (71). However, there is considerable heterogeneity in the clinical presentation of individuals suffering from AUD and comorbid psychiatric disorders, and different patterns of diagnoses necessitate different treatment options. Long-term studies with more complex statistical models are needed in order to better estimate the potential of pharmacological interventions for treating AUD and comorbid diagnoses together. Addressing both AUD and its comorbid psychiatric disorders is crucial in alleviating alcohol-associated pathologies and to achieve better treatment outcomes.

## The Need for Integrated Care Across Medical Specialties and Focus on End-Organ Damage

Approximately 50% of all liver disease mortality is currently attributable to alcohol misuse (72–76), yet there are no FDA approved treatment options for alcohol-associated liver disease (ALD). ALD causes significant morbidity and mortality and is the leading cause of cirrhosis, liver cancer, and acute/chronic liver failure (72–76). Although the pathophysiology of ALD is clearly linked to excessive alcohol consumption, the exact mechanisms remain elusive and span domains of behavior as well as environmental, genetic and epigenetic factors (77, 78). Treatment options for ALD are limited and ultimately include abstinence from alcohol – a goal that is difficult to achieve for most individuals with ALD and/or AUD. Presently, there are only limited pharmacological treatment options available for ALD, which, depending on the degree of liver damage, may include corticosteroids, pentoxifylline and N-acetylcysteine (79–84). Once liver damage progresses to cirrhosis, liver transplant is often the only option. Given the large unmet clinical need for effective new pharmacological interventions for ALD, innovative approaches to identify novel targets and treatments are needed (85). Similarly, alcohol can damage the cardiovascular, metabolic, and gastrointestinal systems and lead to various cancers, all of which contribute significantly to the overall morbidity and mortality of AUD. Given this, pharmacological treatment of AUD cannot be developed isolated from related downstream organ systems affected by alcohol metabolism and comorbidities. Coordination of care between medical specialties is necessary to provide individuals suffering from AUD/ALD integrative care to maximize preventive measures and to minimize adverse health outcomes.

There is also the issue of patient treatment goals and the role of end-organ function and harm reduction endpoints in defining therapeutic success for individual patients. While many clinical trials of new medications and treatment programs for AUD use abstinence as a primary outcome measure, an excessive focus on abstinence as the only relevant outcome for AUD treatment could overshadow treatments that tangibly reduce the harmful physiological effects of drinking; this may also partially explain the failure of several clinical trials despite robust preclinical findings indicating potential in human subjects (86–88). Many treatments are available which facilitate some degree of drinking behavior symptom improvement, and several other medications can treat downstream, end-organ damage resulting from drinking, and these drugs should be given appropriate attention by researchers and clinicians to improve patient outcome when abstinence cannot be achieved.

## Conclusions

AUD is a chronic, often disabling disease with significant morbidity and mortality. Despite non-pharmacological and pharmacological treatment options, most individuals with AUD don't achieve their therapeutic goals, often due to limited efficacy of available treatments. New efforts using precision medicine approaches and novel molecular-based drug discovery efforts are needed to address the complex nature of AUD and its associated psychiatric and end-organ comorbidities.

## Author Contributions

FL designed, drafted, and wrote the manuscript.

## Funding

This work was supported by the National Institutes of Health (NIH) intramural funding [ZIA-AA000242 to FL]; Division of Intramural Clinical and Biological Research of the National Institute on Alcohol Abuse and Alcoholism (NIAAA).

## Conflict of Interest

The author declares that the research was conducted in the absence of any commercial or financial relationships that could be construed as a potential conflict of interest.

## Publisher's Note

All claims expressed in this article are solely those of the authors and do not necessarily represent those of their affiliated organizations, or those of the publisher, the editors and the reviewers. Any product that may be evaluated in this article, or claim that may be made by its manufacturer, is not guaranteed or endorsed by the publisher.
